# Prediction of Effective Strain Distribution in Two-Pass Drawn Wire

**DOI:** 10.3390/ma12233923

**Published:** 2019-11-27

**Authors:** Sang-Kon Lee, In-Kyu Lee, Sung-Min Lee, Sung-Yun Lee

**Affiliations:** Extreme Fabrication Technology Group, Korea Institute of Industrial Technology, 320 Techno sunhwan-ro, Yuga-up, Dalseong-gun, Daegu 42994, Korea; lik1025@kitech.re.kr (I.-K.L.); mytjdals@kitech.re.kr (S.-M.L.); yunskills@kitech.re.kr (S.-Y.L.)

**Keywords:** two-pass wire drawing process, effective strain distribution, strain prediction model, finite element analysis, drawing experiment

## Abstract

In the multi-pass wire drawing process, the diameter of a wire is decreased by continuously passing it through progressively smaller drawing dies. Although the deformation depends on the process variables, in most wire drawing processes, the wire deformation is concentrated on the surface by its direct contact with the drawing dies, causing a nonlinear distribution of radial direction effective strain from the center to the surface. In this study, a new model for predicting this effective strain in two-pass drawn wire was derived based on the upper bound method, and a finite element analysis and drawing experiment were conducted to validate its effectiveness. The proposed model offers a promising approach to determining and thus controlling the strain in multi-pass drawn wire.

## 1. Introduction

The wire drawing process is one of the most common metal forming processes [1]. Drawn wires are widely used in many fields, including in the automotive, construction, chemistry, electric or electronic, and shipbuilding industries. During the wire drawing process, as the wire passes through the drawing die, its length increases and its diameter decreases. In wire drawing process, the non-uniform deformation from center to surface can adversely affect the mechanical properties and straightness of drawn wire and is an important cause of residual stress [2,3]. In particular, the presence of tensile residual stress in drawn wire reduces its yield strength [4] and has deleterious effect on its fatigue and corrosion properties [5]. Therefore, it is important to evaluate the distribution of strain in drawn wire.

In recent years, finite element analysis (FEA) has been frequently used to assess deformation instead of physical experiment. Luksza et al. investigated the distribution of effective strain under different drawing schedules using an FEA [6]. Vega et al. evaluated the deformation of copper wire according to different design parameters varied in an FEA [7]. Additionally, Celentano et al. predicted the distribution of effective strain from the center to the surface of a steel wire based on an FEA [8]. Venet et al. used an FEA to plot the effective strain of a multi-pass steel wire drawing process [9] and observed that the strain distribution showed no sensitivity to the work hardening coefficient of the wire material. Cetlin evaluated the deformation within the drawn aluminum wire cross section using the hardness profile method in two-pass drawing [10]. Cetlin derived the relationship between strain and hardness using the tensile tested specimens and then calculated the strain. In this study, the experiment should be preceded by calculation of the strain. However, if a theoretical model can be derived, the strain will be predicted effectively. In addition, the model will be used to evaluate the mechanical properties of drawn wire.

In this study, a theoretical model, based on the upper bound method, was derived to predict the distribution of radial direction effective strain in two-pass drawn wire. As process parameters such as semi-die angle, reduction ratio, and friction coefficient affect the distribution of strain, the proposed strain prediction model is expressed in terms of these parameters. The proposed model was then applied to predict the distribution of effective strain in a two-pass carbon steel wire drawing process. Then, an FEA was conducted to validate the effective strain predictions. Finally, after a two-pass drawing experiment was conducted and the micro Vickers hardness of the wire was measured across its radius to validate the predicted and simulated strains using the differential deformation between the center and the surface of the drawn wire after each pass.

## 2. Materials and Methods

### 2.1. Material

An AISI 1062 carbon steel wire with an initial diameter of 5.5 mm was used in the two-pass wire drawing process in this study.

### 2.2. Strain Prediction Model

The simulation of metal flow can be used to estimate the deformation load and the average forming pressure when drawing a wire through a die. Among the various methods of analysis for metal forming processes, the upper bound method is the most practical technique when applied to simple deformation processes [11]. This method has been used to predict material flow, forming load, internal defect, collapse failure, and so on [12,13,14,15,16]. However, it has not been used to predict the distribution of strain.

**Figure 1:**
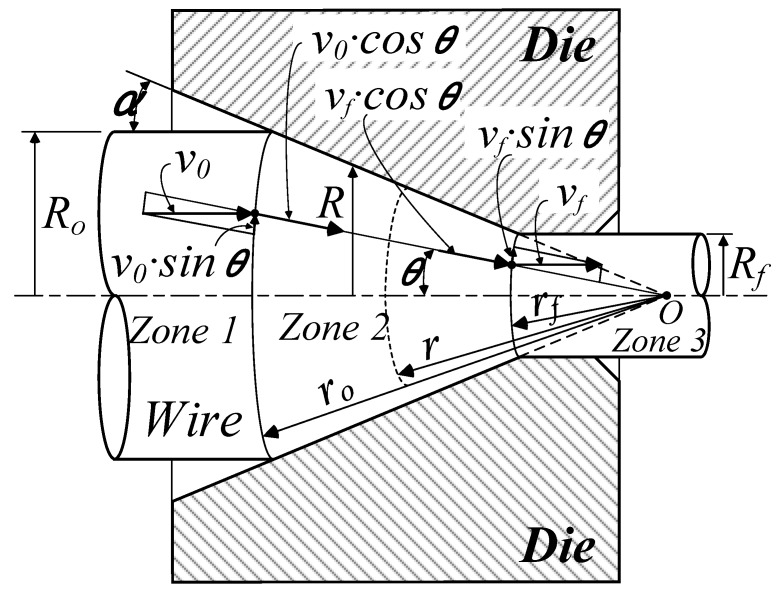
Velocity field of axisymmetric drawing process [17].

In this study, the effective strain of drawn wire was therefore predicted using a kinematically admissible velocity field of the upper bound method, shown for an axisymmetric drawing process in Figure 1 [17]. The effect of generated heat during drawing process was not considered.

**Figure 2:**
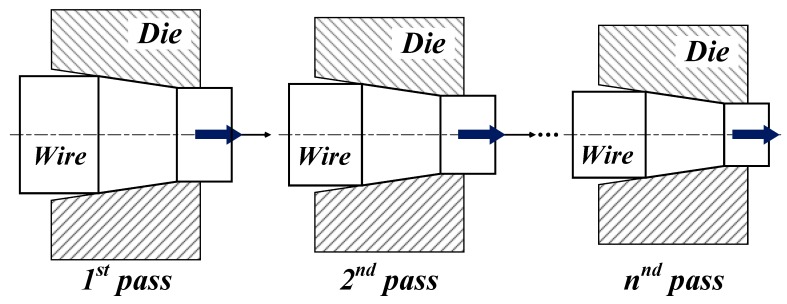
Schematic diagram of multi-pass wire drawing.

Using the spherical coordinate system (*r*, *φ*, *θ*), in the deformation area, the velocity components ${\dot{U}}_{r}$, ${\dot{U}}_{\theta }$, and ${\dot{U}}_{\varphi }$ of an arbitrary location *r* from center *O* can be written as:

(1) $$\begin{array}{c} {\dot{U}}_{r}=v=-v_{f}\cdot r_{f}^{2}\cdot \frac{cos\theta }{r^{2}}\\ {\dot{U}}_{\theta }={\dot{U}}_{\varphi }=0 \end{array}$$

where *v_f_* is the velocity at the die exit, *r_f_* is the distance from *O* to the spherical boundary between Zone 2 and Zone 3, and *θ* is the angle defined in Figure 2. From Equation (1), the strain rate components can be defined as:

(2) $$\begin{array}{c} {\dot{\varepsilon }}_{r}=\frac{\partial {\dot{U}}_{r}}{\partial r}=2v_{f}\cdot r_{f}^{2}\cdot \frac{cos\theta }{r^{3}}\\ {\dot{\varepsilon }}_{\theta }=\frac{{\dot{U}}_{r}}{r}=-v_{f}\cdot r_{f}^{2}\cdot \frac{cos\theta }{r^{3}}\\ {\dot{\varepsilon }}_{\varphi }=\frac{{\dot{U}}_{r}}{r}=-v_{f}\cdot r_{f}^{2}\cdot \frac{cos\theta }{r^{3}} \end{array}$$

Next, assuming axial cylindrical symmetry, the shear strain rate components can be derived as follows:

(3) $$\begin{array}{c} {\dot{\varepsilon }}_{r\theta }=\frac{1}{2r}\frac{\partial }{\partial U_{r}}=\frac{1}{2}v_{f}\cdot r_{f}^{2}\cdot \frac{sin^{2}\theta }{r^{3}}\\ {\dot{\varepsilon }}_{\theta \varphi }={\dot{\varepsilon }}_{r\varphi }=0 \end{array}$$

The effective strain rate ($\dot{\bar{\varepsilon }}$) is accordingly given by:

(4) $$\dot{\bar{\varepsilon }}=\sqrt{\frac{2}{3}{\dot{\varepsilon }}_{ij}{\dot{\varepsilon }}_{ij}}≅2v_{f}\cdot r_{f}^{2}\cdot \frac{1}{r^{3}}\sqrt{1-\frac{11}{12}sin^{2}\theta }$$

when $\dot{\bar{\varepsilon }}$ is a function of $\dot{\varepsilon }$ ($\dot{\bar{\varepsilon }}=\dot{\varepsilon }\left(r,\;\theta \right)$). At the die exit, the total strain is calculated by integrating the strain rate over the distance *r*. Therefore, the effective strain can be express as:

(5) $$\bar{\varepsilon }=\int_{t=0}^{t}\dot{\bar{\varepsilon }}dt$$

where,

(6) $$\mathrm{dt}=\frac{dr}{v}=-\frac{v^{2}dr}{v_{f}\cdot r_{f}^{2}\cdot cos\theta }$$

Therefore, inserting Equations (4) and (6) into Equation (5), the effective strain can be calculated by Equation (7).

(7) $$\begin{array}{c} \bar{\varepsilon }=-2\cdot \frac{\sqrt{1-\frac{11}{12}\sin^{2}\theta }}{\cos\theta }\int_{r=0}^{r}\frac{dr}{r}\\ =2\cdot \frac{\sqrt{1-\frac{11}{12}sin^{2}\theta }}{cos\theta }\cdot ln\left(\frac{r_{0}}{r}\right) \end{array}$$

From Figure 1 and Equation (7), it can be observed that the maximum effective strain will occur on the surface of the wire when θ is equal to *α*. Because *α* is the maximum value of θ. It can be observed that the effective strain increases with the increase in the reduction ratio and the semi-die angle.

After being drawn, i.e., when *r* is changed to *r_f_*, the accumulated effective strain is calculated by:

(8) $$\bar{\varepsilon }=2\cdot \frac{\sqrt{1-\frac{11}{12}sin^{2}\theta }}{cos\theta }\cdot ln\left(\frac{R_{0}}{R_{f}}\right)$$

where *r_0_/r_f_* = *R_o_*/*R_f_*.

When *r* is *r_f_*, *sinθ* = *R*/*R_f_* = *R*/*R_f_* ·sin*α*. Therefore, from Equation (8), the effective strain can be expressed in the following Equation (9).

(9) $$\bar{\varepsilon }=2\cdot \frac{\sqrt{1-\frac{11}{12}{\left(\frac{R}{R_{f}}\right)}^{2}sin^{2}\alpha }}{\sqrt{1-{\left(\frac{R}{R_{f}}\right)}^{2}sin^{2}\alpha }}\cdot ln\left(\frac{R_{0}}{R_{f}}\right)$$

In Equation (9), the effective strain increases with the increase in the reduction ratio. At a small semi-die angle, the strain is constant through the wire radius. As the semi-die angle increases, so does the effective strain, and the increase in the effective strain on the surface is relatively higher than that in the center.

If *α* is nearly zero, there is no redundant deformation in the wire. Therefore, Equation (9) can be written as the ideal average effective strain as follows:

(10) $${\bar{\varepsilon }}_{avg}=2\cdot ln\left(\frac{R_{0}}{R_{f}}\right)$$

It is important to note that friction has a considerable effect on the outcome of the wire drawing process. However, Equation (9) cannot consider the effect of friction. Cetlin described the distribution of strain from center to surface using quadratic a function [10]. Therefore, in this study, in order to consider the effect of friction on the distribution of the effective strain, the quadratic function of friction was added to Equation (9) [18]:

(11) $$\bar{\varepsilon }=2\cdot \frac{\sqrt{1-\frac{11}{12}\left(\frac{R}{R_{f}}\right)sin^{2}\alpha }}{\sqrt{1-{\left(\frac{R}{R_{f}}\right)}^{2}sin^{2}\alpha }}\cdot ln\left(\frac{R_{0}}{R_{f}}\right)+\mu \cdot {\left(\frac{R}{R_{f}}\right)}^{2}$$

where the *μ* term accounts for the effects of friction.

As shown in Figure 2, wire continuously passes through the progressively smaller drawing dies during a multi-pass wire drawing process. Therefore, the total strain is accumulated in the wire as the pass progresses according to:

(12) $${\bar{\varepsilon }}_{n}=\sum_{i=1}^{n}{\bar{\varepsilon }}_{i}$$

where *n* is the number of passes.

### 2.3. Two-Pass Wire Drawing Pass Schedule

In this study, a two-pass wire drawing process consisting of two-passes was used to apply the prediction model proposed in Section 2.2. The initial and the final diameters of the wire were 5.50 mm and 4.00 mm, respectively, representing a total reduction ratio of 47.11%. Table 1 shows the pass schedule of the two-pass drawing process used in this study.

### 2.4. Finite Element Analysis

**Figure 3:**
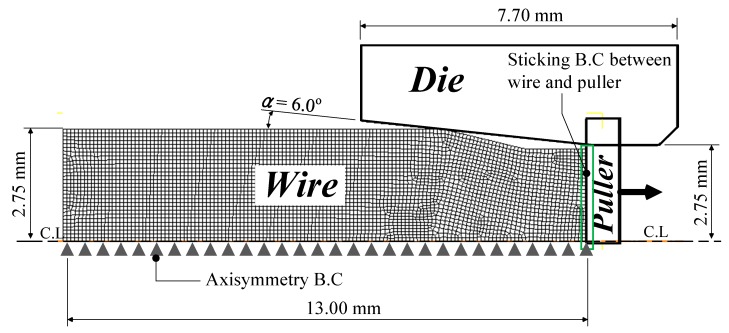
Initial FEA model.

An FEA was performed in this study to verify the proposed prediction model. The DEFORM 2D software (Ver. 11.2, SFTC, Columbus, OH, USA) was used to conduct the analysis. Figure 3 shows the initial analysis model. The wire was defined as a rigid-plastic material and the other tools as rigid bodies [19]. The analysis conditions including friction coefficient [20] are summarized in Table 2. In the analysis, the deformation history of the previous pass was considered to analyze the next pass during the two-pass drawing process.

### 2.5. Wire Drawing Experiment

**Figure 4:**
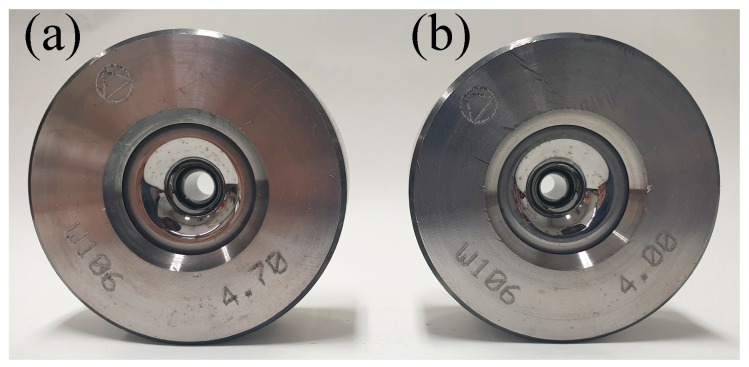
Drawing dies for the (**a**) first pass and (**b**) second pass.

**Figure 5:**
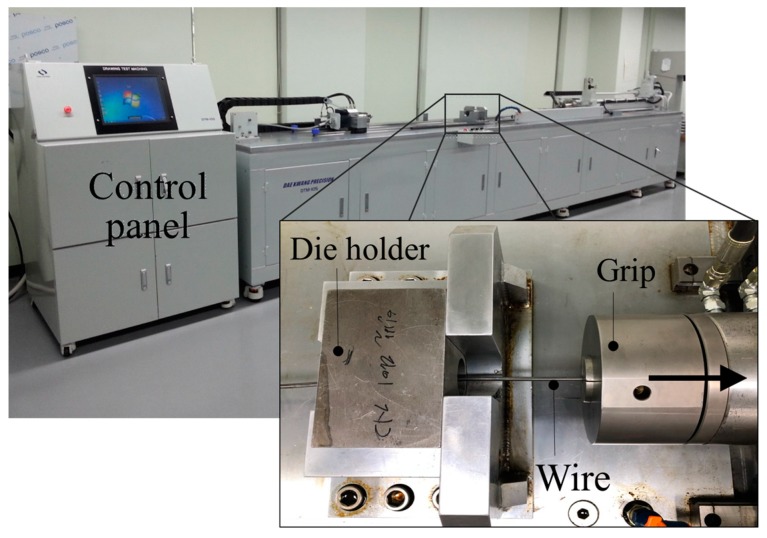
Precision drawing machine.

In order to validate the effectiveness of both the proposed prediction model and the FEA results, a two-pass wire drawing experiment was also performed. Figure 4 and Figure 5 show the drawing dies and the precision drawing machine, respectively, used in the experiment.

**Figure 6:**
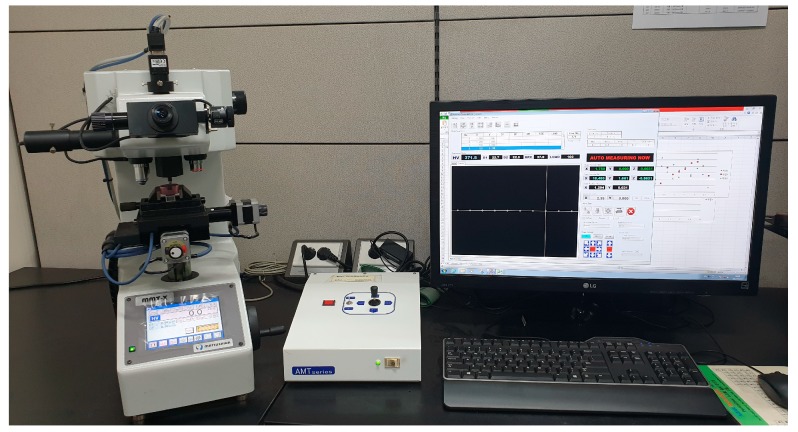
Micro-hardness testing machine.

After the two-pass drawing process, the micro Vickers hardness of the drawn wire was measured for comparison with the results of the prediction model and the FEA. Figure 6 shows the automatic micro-hardness testing machine (AMT-X7FS Type B, Matsuzawa Co.,Ltd, Akita-shi, Japan) used in this study.

## 3. Results and Discussion

### 3.1. Material Properties

**Figure 7:**
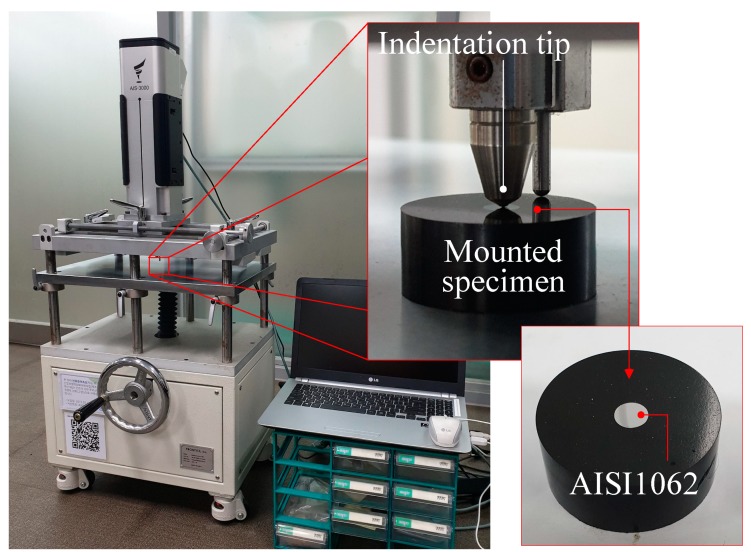
Indentation test for flow stress curve.

The flow stress curve used in the FEA was obtained through indentation tests using a non-destructive indenter (AIS3000, Frontics Inc., Seoul, Korea) [21,22,23], shown in Figure 7. The specimen dimensions for indentation testing were *φ*5.5 × *h*12.0 mm. Prior to indentation testing, the specimen surface was finely polished with 1.0 μm Al_2_O_3_ powder [22].

**Figure 8:**
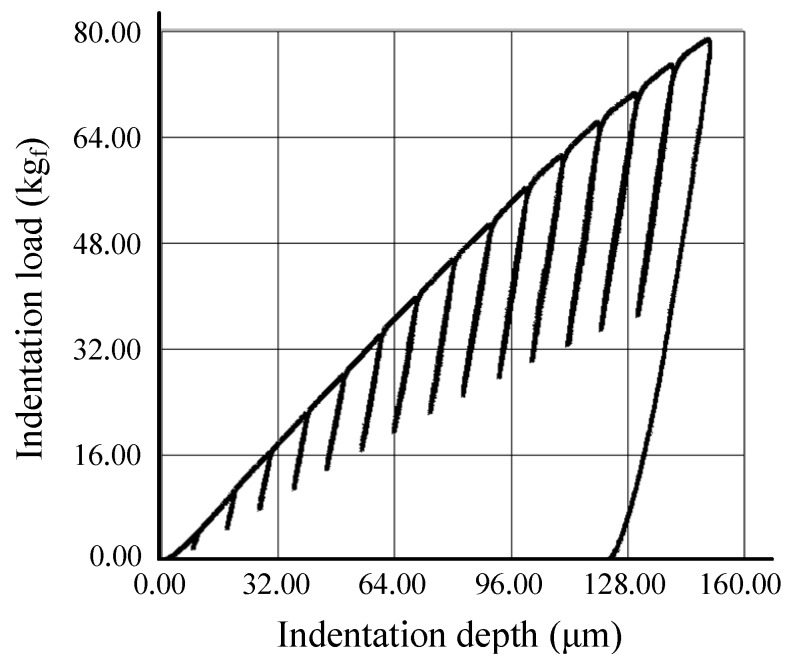
Measured indentation load-depth curve.

**Figure 9:**
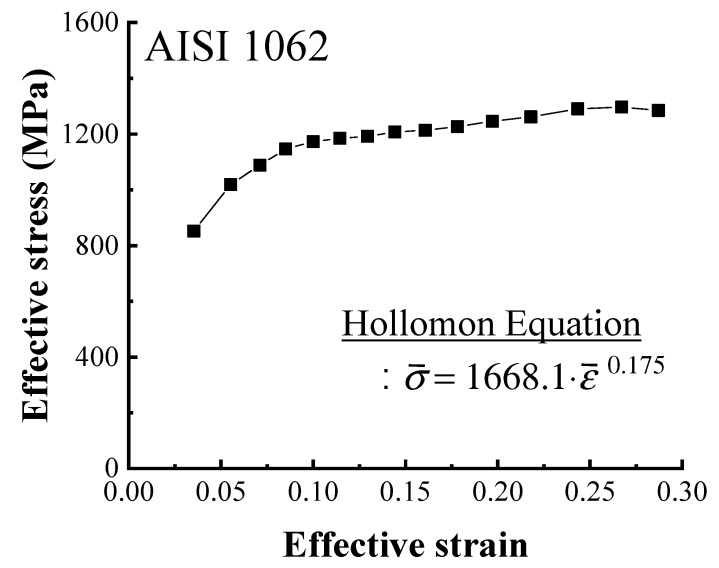
Flow stress curve of the initial wire.

The indentation tests were conducted five times to ensure reproducibility. One of the measured indentation load-depth curves of the initial wire is shown in Figure 8. The results of these indentation tests were used to obtain the flow stress curve of the initial wire [21], shown in Figure 9, with the Hollomon equation derived from the curve. This equation was used to define the material behavior in the FEA. The other material properties obtained from the indentation test were summarized in Table 3.

### 3.2. Result of Finite Element Analysis

**Figure 10:**
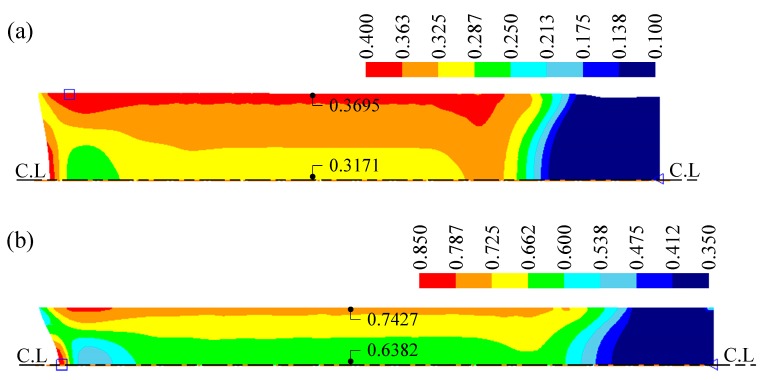
Distribution of effective strain after the (**a**) first pass and (**b**) second pass.

Figure 10 shows the distribution of effective strain after each pass. The strain increases gradually with pass number as the wire diameter decreases. After both passes, the maximum strain can be observed to be near the surface of the wire. The difference in strain between the center and near-surface increases with pass number because of the increase in non-uniform deformation: The maximum differences in the strain between the center and near-surface were 0.0524 and 0.1045 after the first and second pass, respectively.

### 3.3. Comparison of Prediction Model and Finite Element Analysis

**Figure 11:**
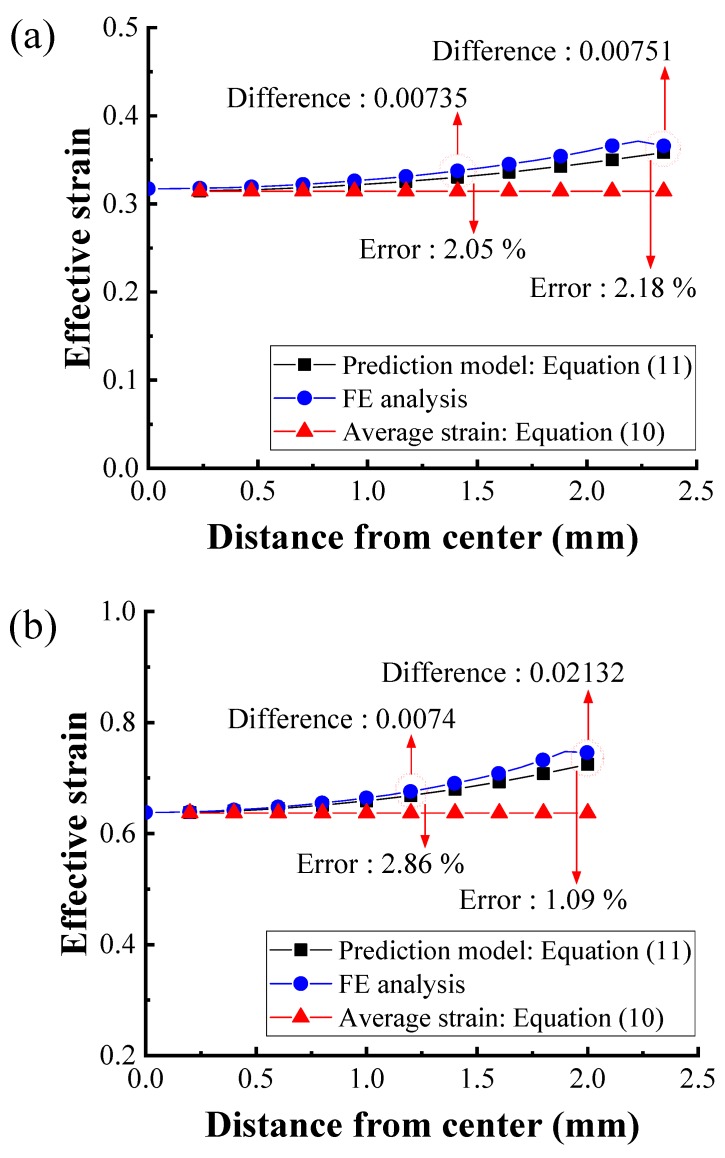
Comparison of effective strain after the (**a**) first pass and (**b**) second pass.

Figure 11 shows a comparison of strain as determined by the average strain in Equation (10), the proposed prediction model including friction in Equation (11), and the FEA after each pass. The strain can be observed to increase from the center to the surface because of the direct contact between die and wire after each pass. Furthermore, the proposed model and FEA provide different strains at the mid-radius, and surface of the wire after each pass: at the mid-radius this difference is 0.00703 and 0.01084 after the first and second pass, respectively; at the surface, this difference is 0.00947 and 0.01764 after the first and second pass, respectively. According to the proposed prediction model, the maximum difference between the strain in the center and near the surface were 0.04352 and 0.00889 after the first and second pass, respectively. The maximum error is less than 3%. These differences coincide with those resulting from the FEA. Additionally, the average strain can be observed to best match the center strains determined by the prediction model and the FEA, whereas it under-estimates the strain at the mid-radius and surface of the wire compared with the results of the prediction model and FEA. This is likely because the average strain calculation model (Equation (10)) does not consider the surface deformation caused by the direct contact between the die and wire.

Cetlin suggested a parabolic equation for the distribution of strain using an experiment for establishing the relationship between hardness and strain [10]. In Figure 11, it can be observed that the distribution of strain is similar not only to that of Cetlin’s research but also to that of Vent’s research using FEA [9].

### 3.4. Hardness of Drawn Wires

**Figure 12:**
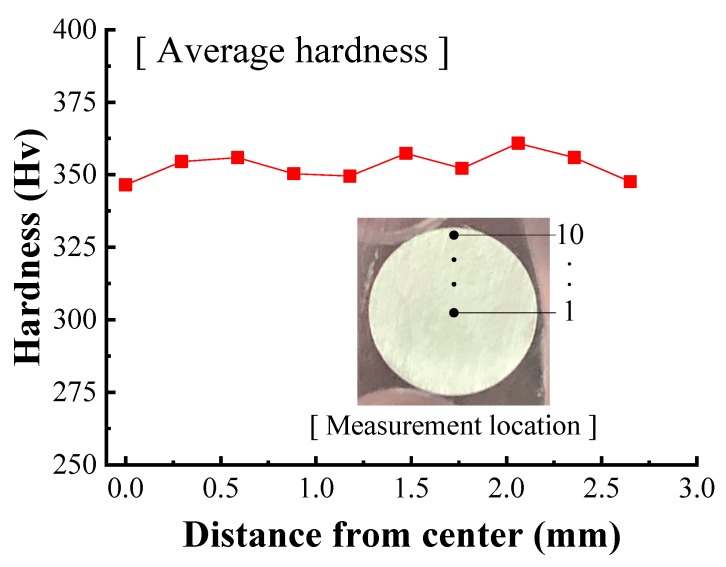
Hardness of the initial wire.

Before and after each pass of the two-pass drawing experiment, the hardness of the wire was measured at ten locations along the radius from the center to the surface of three wire specimens to indirectly evaluate the strain. A 100 g load was applied for measuring the hardness. Figure 12 shows the average measured hardness at each location in the initial wire, in which it can be observed that there is little difference in hardness across the cross section. The average hardness of the initial wire was 353.06 Hv.

**Figure 13:**
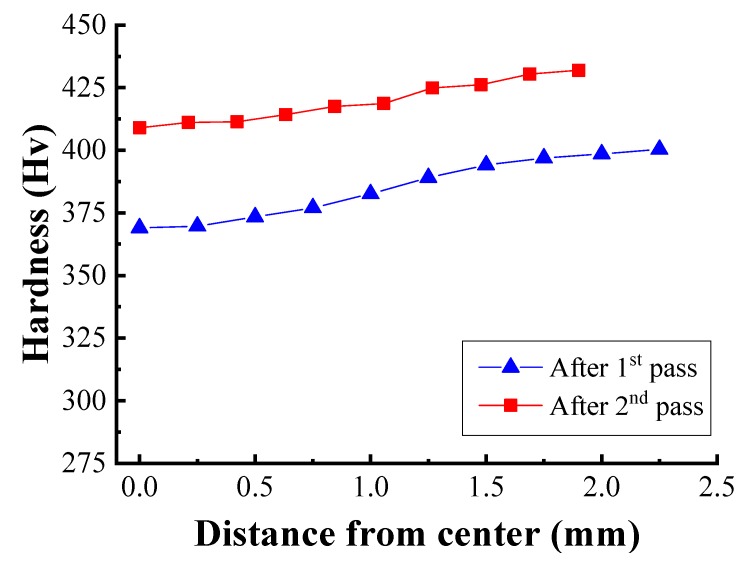
Hardness profile at each pass.

The hardness of the wire increased during the metal forming process due to the strain hardening effect. Figure 13 shows the measured hardness of the drawn wire before and after each pass, in which it can be observed that the hardness increases from the center to the surface with progressive drawing passes due to the higher deformation near the surface of the wire. Critically, the distributions in Figure 13 are similar to the strain distributions in Figure 11. This result is the same result as in Cetlin’s research using [10].

**Figure 14:**
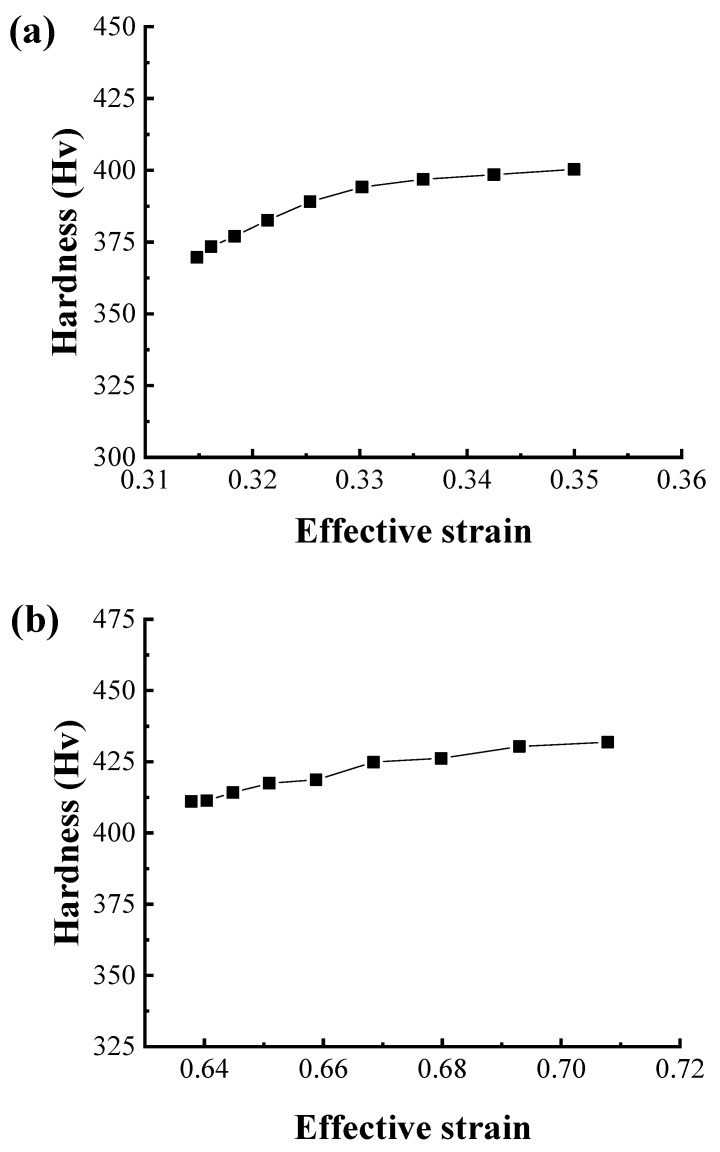
Relationship between strain and hardness after the (**a**) first pass and (**b**) second pass.

Figure 14 depicts the relationship between the strain and the hardness of the drawn wire, in which it can be observed that the hardness increases with the increase in strain. After the first pass, the hardness increases less with increasing strain, whereas, after the second pass, the hardness increases relatively uniformly with increasing strain because the strain has increased more on the surface than in the center.

## 4. Conclusions

The objective of this study is to propose a theoretical strain prediction model in drawn wire. In general, the distribution of strain is not uniform but rather gradually increases from the center to the surface because of the direct contact between the wire surface and the die.

Using a kinematically admissible velocity field of the upper bound method, a strain prediction model was proposed for two-pass drawn wire. The strain was calculated from the center to the surface of a drawn wire using the prediction model under a two-pass drawing process that reduced the wire diameter from 5.50 mm to 4.70 mm to 4.0 mm. The flow stress curve of the AISI 1062 wire was then determined using indentation tests and used to perform FEA. The results of the FEA were compared to the strain predicted by the proposed model over two-passes. The differences in the strain on the surface of the drawn wire were 0.00947 and 0.01764 after the first and second passes, respectively. Although there was very little difference, the strains coincided with each other well at each pass. Moreover, it can be observed that the average strain was under-estimated compared with the result of the prediction model and FEA. Finally, the micro-Vickers hardness of a series of drawn wires was measured after two-pass wire drawing experiment to evaluate the strain distribution, finding that the hardness profile was similar to the predicted strain profile: The hardness increased with the increase in strain. While the proposed prediction model was found to agree well with the FEA result, as it is difficult to directly measure strain in drawn wire, further research remains necessary to establish a relationship or empirical model between strain and hardness. Using this relationship, it will be possible to quantitatively compare strain and hardness and thus to accurately evaluate the accumulated strain in drawn wire based on measured hardness. It can be considered to be a more efficient method compared to previous studies because the distribution of strain can be predicted by the proposed prediction model.

## Figures and Tables

**Table 1:** Pass schedule of two-pass drawing process.

Pass No.	Inlet Dia. (mm)	Exit Dia. (mm)	Semi-Die Angle (°)	Reduction (%)
1	5.50	4.7	6.0	29.96
2	4.7	4.0	6.0	27.57

**Table 2:** FEA conditions.

Conditions	Value
No. of elements	2539
No. of nodes	2661
Friction coefficient (*μ*) (Phosphate coating)	0.06
Semi-die angle (°)	6.0
Bearing length (mm)	0.3D_in_ (D_in_: inlet diameter of wire)
Drawing velocity (mm/s)	1.0

**Table 3:** Mechanical properties of the initial wire.

Properties	Value
Yield strength (MPa)	570.0
Tensile strength (MPa)	1206.5
